# Mapping University-Based Master of Public Health Programs in the Arab world

**DOI:** 10.5334/aogh.3297

**Published:** 2021-07-20

**Authors:** Iman Nuwayhid, Ghida Krisht, Samer Jabbour, Jocelyn DeJong, Huda Zurayk

**Affiliations:** 1 Faculty of Health Sciences, American University of Beirut, Beirut, Lebanon

## Abstract

**Background:**

The Arab world faces numerous health challenges that mandate a competent public health workforce and strengthening public health education.

**Objective:**

To analyze university-based Master of Public Health (MPH) programs offered at Faculties of Public Health (FPH) and of Medicine (FM) in Arab countries.

**Methods:**

We searched a regional database of academic public health institutions, conducted a search of university websites, and reviewed websites of the Association of Arab Universities and World Directory of Medical Schools. A factsheet for each MPH program was emailed to deans of respective faculties for validation and completion. We examined associations between presence of such programs and population size and Human Development Index (HDI).

**Findings:**

A total of 19 FPH and 10 FM at 28 universities offer MPH programs (7 programs per 100 million population). Ten countries offer no MPH programs; the remaining 12 offer 1–5 programs each. Ten MPH programs were initiated over 45 years (1965–2009) and another 19 over 10 years (2010–2019). No correlation was observed between offering an MPH program and the country’s HDI or population size. Less than half of the programs admit students from fields outside health. FPH and FM-based programs are comparable in offering core disciplines but FPH programs offer more Social and Behavioral Sciences (83% vs. 60%). More FM-based programs provide practicum training (78% vs. 53%); 10 programs offer none. Epidemiology, alone or with Biostatistics, and Health Management and Policy are the two most frequently offered MPH concentrations. None of the MPH programs offer a concentration on public health in conflict or humanitarian crises; only one offers a certificate on the theme. Only three programs, all FPH-based, reported international accreditation.

**Conclusions:**

The recent increase in MPH programs in Arab countries is encouraging. Critical gaps are absence of MPH programs in 10 countries, less coverage of the social sciences, and lack of practicum experience in 10 programs. Upgrading and promoting public health education across the region to fill these gaps calls for collaboration among existing MPH programs. More in-depth analysis of the history and mission of these programs, as well as their admission criteria and curricula, is needed.

## Introduction

The 22 country members of the Arab League were inhabited by 428 million people in 2019 [1]. These include some of the wealthiest and poorest countries globally [2]. Many countries endure a high burden of non-communicable diseases and injuries, while others are still also burdened by diseases of poverty [3,4,5]. Variability exists within and between countries reflecting socio-economic and health service inequalities. The region has experienced multiple protracted conflicts, hosts the world’s highest number of displaced and refugee populations [3], and faces serious environmental challenges [7,8]. The COVID-19 pandemic has exposed the lack of public health preparedness in most countries [4].

This complex picture mandates a competent public health workforce and calls for strengthening public health education in all countries of the region [10,11,12,13], including through launching standalone schools that recognize the comprehensive, multidisciplinary and autonomous nature of public health.

This paper presents a first mapping of university-based Master of Public Health (MPH) programs in the Arab region and describes their historical growth, geographical distribution, course content, research and practical training, and other significant features analyzed according to the type of hosting faculty (public health or medicine).

## Methods

We used a multi-pronged search strategy to identify MPH programs and variations in them (e.g., Master of Science in Public Health or Master of Public and Global Health), initiated before January 1, 2020, in two sets of university faculties: 1) standalone faculties/schools/institutes of public health or health sciences (FPH); and 2) medical faculties/schools/colleges (FM) with departments of public health or community medicine that may offer MPH programs.

We searched a regional database of public health institutions, programs and degrees conducted for the Eastern Mediterranean Region Academic Institutions’ Network in 2012 [14], and updated in 2015 and 2017. We conducted a comprehensive internet search using an expanded list of keywords (“public health,” “academic,” “degrees,” “programs,” university,” country name,” “Arab,” “education,” “Master in Public Health,” “MSc in Public Health,” “Master en Santé Publique”), and searched the websites of Association of Arab Universities and the World Directory of Medical Schools [15,16].

For each MPH program we developed a factsheet (Appendix 1) based on information gathered from websites, and emailed it to deans of respective faculties for validation and completion concerning governance and the number of graduates in the last two years. We received responses from 26 out of 29 programs (90%).

We used Spearman correlation tests to examine the relationship between presence of at least one MPH program in a country and its Human Development Index and population size (coded into low, medium, and high ranges) [17,18].

We grouped the Arab countries under four sub-regions: Maghreb (Algeria, Libya, Mauritania, Morocco, and Tunisia); Nile basin (Comoros, Djibouti, Egypt, Somalia, and Sudan); Mashreq (Iraq, Jordan, Lebanon, occupied Palestinian territory [oPt], and Syria); Gulf (Bahrain, Kuwait, Oman, Qatar, Saudi Arabia, UAE, and Yemen).

The study was not submitted for ethical clearance since it is limited to a review of published information and not a study of human subjects.

## Results

A total of 19 FPH and 10 FM at 28 universities offer MPH programs (***Tables 1a*** and ***1b***).

Table 1List of Faculties (listed in chronological order) offering MPH programs in Arab countries, 2019.

**Table 1a:** Faculties of Public Health offering MPH programs, 2019.


COUNTRY	UNIVERSITY	PRIVATE/ PUBLIC	FACULTY OF PUBLIC HEALTH (FPH)	YEAR OF FPH ESTABLISHMENT	YEAR OF MPH INITIATION

Sudan	University of Khartoum	Public	Faculty of Public and Environmental Health	1933	1991

Lebanon	American University of Beirut	Private	Faculty of Health Sciences	1954	1971

Egypt	Alexandria University	Public	High Institute of Public Health	1956	1968

Sudan	Ahfad University for Women	Private	School of Health Sciences	1966	2013

oPt	Birzeit University	Private	Institute of Community and Public Health	1978	1996

Lebanon	Lebanese University	Public	Faculty of Public Health	1981	2010

oPt	Al-Quds University	Public	Faculty of Public Health	1994	1997

Lebanon	University of Balamand	Private	Faculty of Health Sciences	1995	2015

Lebanon	Jinan University	Private	Faculty of Public Health	1999	2014

Saudi Arabia	King Saud Bin Abdulaziz University for Health Sciences	Public	College of Public Health and Health Informatics	2006	2015

UAE	Hamdan Bin Mohammed Smart University	Semi-private	School of Health and Environmental Studies	2009	2011

Qatar	Qatar University	Public	College of Health Sciences	2012	2015

Somalia	Amoud University	Public	College of Health Sciences	2012	2012

Kuwait	Kuwait University of Health Sciences	Public	Faculty of Public Health	2013	2018

Somalia	Benadir University	Private	Faculty of Health Sciences/ School of Postgraduate Studies	2014	2014

Morocco	Université Mohammed VI - Des Sciences de la Santé	Private	International School of Public Health	2014	2014

Saudi Arabia	Imam Abdulrahman Bin Faisal University^a^	Public	College of Public Health	2015	2015

Egypt	American University in Cairo	Private	Institute of Global Health and Human Ecology	2018	2019

Somalia	Mogadishu University	Private	Information not available, neither by website nor by direct contact		


oPt: occupied Palestinian territory. ^a^ Not validated by response to the fact sheet.

**Table 1b:** Faculties of Medicine offering MPH programs, 2019.


COUNTRY	UNIVERSITY	PRIVATE/ PUBLIC	FACULTY OF MEDICINE (FM)	UNIT AT FM OFFERING MPH	YEAR OF FM ESTABLISHMENT	YEAR OF MPH INITIATION

Lebanon	Saint Joseph University of Beirut	Private	Faculty of Medicine	Higher Institute of Public Health	1883	2016

Jordan	University of Jordan	Public	Faculty of Medicine	Department of Family and Community Medicine	1971	2000

Kuwait	Kuwait University of Health Sciences	Public	Faculty of Medicine	Department of Community Medicine and Behavioral Sciences	1973	2013

Jordan	Jordan University of Science and Technology	Public	Faculty of Medicine	Department of Public Health and Community Medicine	1984	1999

UAE	UAE University	Public	College of Medicine and Health Sciences	Institute of Public Health	1984	2010

Sudan	University of Medical Sciences and Technology	Private	Faculty of Medicine	Department of Public and Tropical Health	1996	2002

UAE	Gulf Medical University	Private	College of Medicine	Department of Community Medicine	1998	2010

oPt	An-Najah National University	Public	Faculty of Graduate Studies	Medical and Health Sciences Program	1999	1999

Saudi Arabia	Alfaisal University	Private	College of Medicine	MPH Program	2002	2016

Mauritania	University of Nouakchott^a^	Public	Faculty of Medicine	MPH Program	2006	2008


oPt: occupied Palestinian territory. ^a^ Not validated by response to the fact sheet.

### Geographic distribution of MPH programs

**Figure 1:**
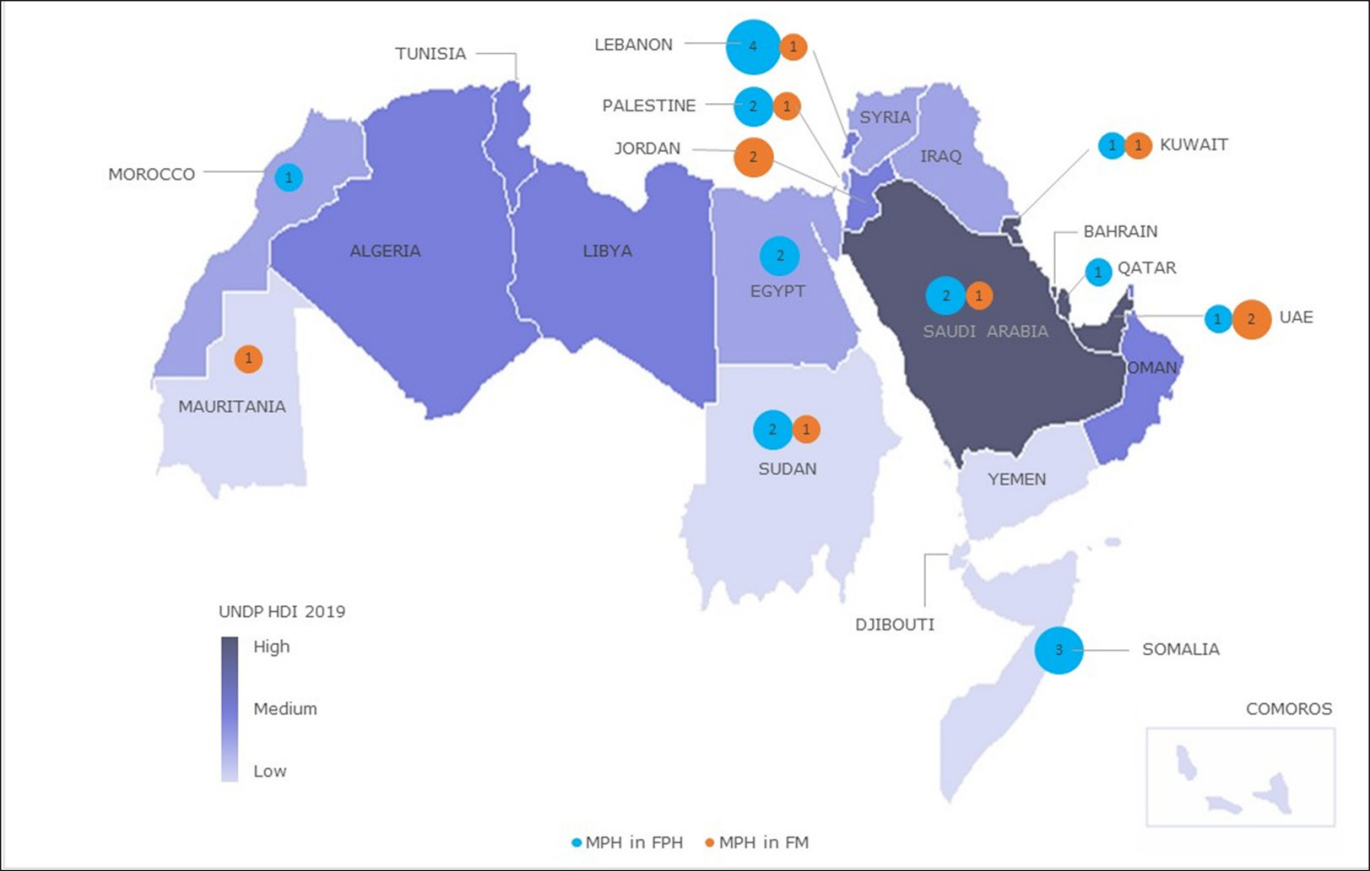
MPH programs in Faculties of Public Hralth (FPH) and Medicine (FM) in Arab Countries, 2019. *Note:* The map is superimposed on a UNDP heatmap of Arab countries by Human Development Index.

Ten countries across the Arab world’s four sub-regions offer no programs (***Figure 1***): Maghreb (Algeria, Libya, Tunisia), Nile Basin (Comoros, Djibouti), Mashreq (Iraq, Syria), and Gulf (Bahrain, Oman, Yemen). For the 12 countries offering at least one program, the number of programs per country and the type of host faculty varied. While Lebanon, one of the smaller countries of the region, offers five programs, other countries offer three (oPt, Saudi Arabia, Somalia, Sudan, United Arab Emirates [UAE]), two (Egypt, Jordan, Kuwait), or one (Mauritania, Morocco, Qatar). Programs are offered at both FPH and FM in six countries, only at FPH in four countries, and only at FM in two countries. The average number of programs per 100 million population for the 22 Arab states is seven (range 0–73) (***Table 2***).

**Table 2:** Number of MPH programs (end 2019) and MPH graduates (2018 and 2019) in Arab countries.


COUNTRY	HDI^A^	POPULATION^B^	MPH PROGRAMS PER 100 MILLION POPULATION	MPH PROGRAMS IN EACH COUNTRY DISTRIBUTED BY:			NUMBER OF GRADUATES FROM MPH PROGRAMS THAT GRADUATED STUDENTS AND REPORTED THE DATA			

				DID NOT REPORT GRADUATES	DID NOT GRADUATE ANY STUDENTS	REPORTED AND GRADUATED STUDENTS	AY 2017–2018	AY 2018–2019	AVERAGE PER YEAR	AVERAGE PER 10 MILLION POPULATION^C^

Egypt	0.700	100,388,073	2		1	1	50	50	50	5.0

Algeria	0.759	43,053,054	0							

Sudan	0.507	42,813,238	7			3	93	118	105.5	24.6

Iraq	0.689	39,309,783	0							

Morocco	0.676	36,471,769	3			1	60	60	60	16.5

Saudi Arabia	0.857	34,268,528	9	2	1		Not provided	Not provided		

Yemen	0.463	29,161,922	0							

Syria	0.549	17,070,135	0							

Somalia	N/A	15,442,905	19	1		2	46	84	65	42.1

Tunisia	0.739	11,694,719	0							

Jordan	0.723	10,101,694	20			2	25	32	28.5	28.2

UAE	0.866	9,770,529	31			3	16	31	23.5	24.1

Lebanon	0.757	6,855,713	73			5	115	105	110	160.5

Libya	0.708	6,777,452	0							

Oman	0.834	4,974,986	0							

oPt	0.690	4,685,306	64			3	99	61	80	170.7

Mauritania	0.527	4,525,696	22			1	20	20	20	44.2

Kuwait	0.808	4,207,083	48		1	1	4	3	3.5	8.3

Qatar	0.848	2,832,067	35			1	10	9	9.5	33.5

Bahrain	0.838	1,641,172	0							

Djibouti	0.495	973,560	0							

Comoros	0.538	850,886	0							

Total		427,870,270	7	3	3	23	538	573	555.5	14.1


oPt: occupied Palestinian territory. ^a^ UNDP Human Development Index (HDI) Ranking 2019, *http://hdr.undp.org/en/content/2019-human-development-index-ranking*. ^b^ World Bank Population Data 2019, *https://data.worldbank.org/indicator/sp.pop.totl*. ^c^ The total population denominator (393,601,742) excludes Saudi Arabia.

We found no correlation between having at least one program in a country and its level of human development (ρ = 0.297, p-value = 0.18) or population size (ρ = 0.155, p-value = 0.50) (***Table 2***). The number of graduates per country for the two last years, is available for 26 programs (90%) and missing for Saudi Arabia (***Table 2***). These programs generate 555 new graduates per year or, on average, 14.1 graduates per year per 10 million population, with wide variation between countries (5–171). Some of the smallest countries, such as Lebanon and oPt, generate the largest number of graduates per 10 million (160, 171, respectively), while some of the larger countries, such as Egypt and Morocco, generate substantially fewer graduates (5, 16.5, respectively).

### Historical trends

**Figure 2:**
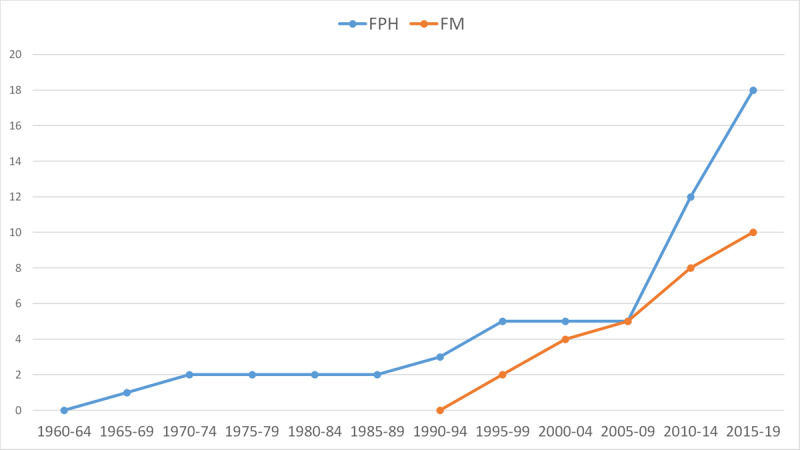
Cumulative number of MPH programs initiated in Arab countries by Faculty, 1960–2019.

Examining the cumulative number of MPH programs established in five-year periods between 1965 and 2019 (***Figure 2***), shows MPH programs were initiated in FPH 30 years before their initiation in FM. The programs at the High Institute of Public Health (HIPH) at the University of Alexandria, Egypt, initiated in 1968, and at the Faculty of Health Sciences at the American University of Beirut (AUB), initiated in 1971, were the only two MPH programs over a period of 20 years (***Table 1a***). Three more programs were established in the 1990s, with 2010–2019 showing the most intense growth of FPH programs increasing to 18. At FM, the first two MPH programs were launched in 1999 at the Jordan University for Science and Technology, Jordan, and An-Najah National University, oPt. The cumulative number of MPH programs at FM grew steadily and gradually but remained lower than the number of programs in FPH.

**Figure 3:**
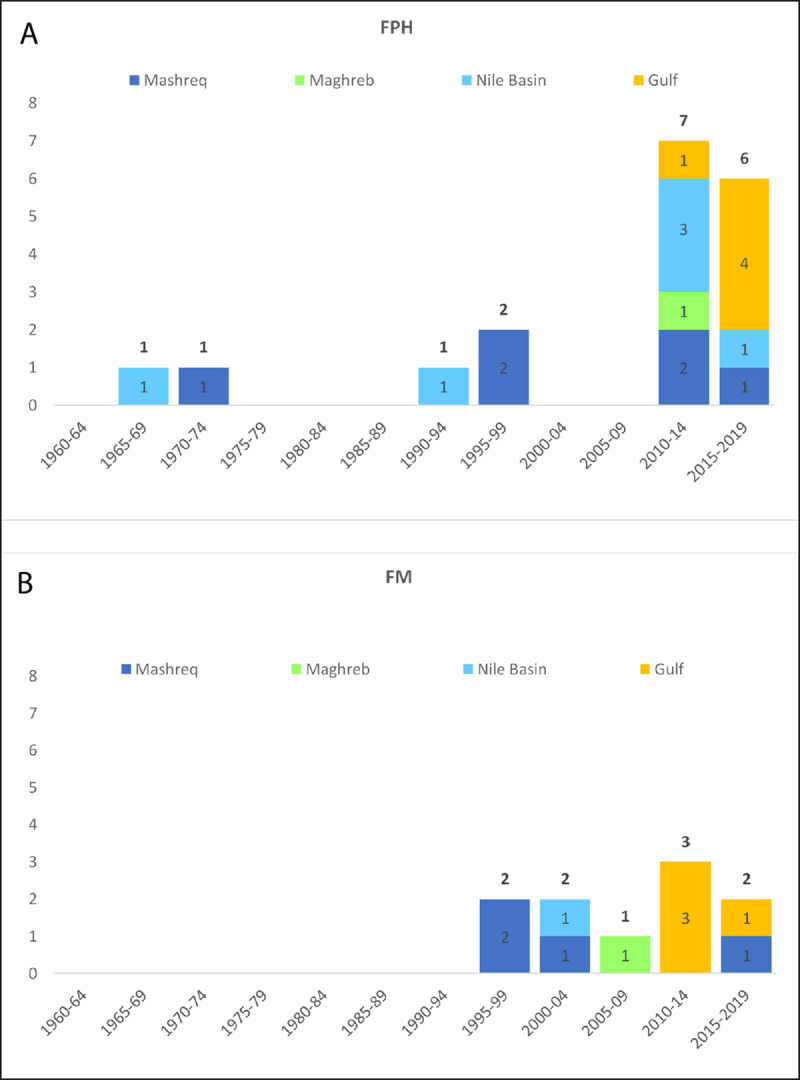
**(A)** Number of new MPH programs initiated in Arab countries by Faculty, period, and sub-region, 1960–2019: New MPH programs in Faculties of Public Health (FPH) by period and sub-region. **(B)** Number of new MPH programs initiated in Arab countries by Faculty, period, and sub-region, 1960–2019: New MPH programs in Faculties of Medicine (FM) by period and sub-region.

***Figure 3A*** and ***3B***. shows the initiation of new MPH programs by year and sub-region in FPH and FM, respectively. Initiation of new programs was highest after 2009 (13 FPH, 5 FM), with 50% of these programs initiated at universities in the Gulf (5/13 in FPH and 4/5 in FM).

### Features of MPH programs

The 29 programs are almost equally distributed between public and private universities, 9/19 in FPH and 6/10 in FM at public universities (***Table 1***) with 1/1 in Maghreb, 3 public/5 private in the Nile basin, 5/5 in Mashreq with 4/5 programs in private universities in Lebanon, and 6 public/3 private in the Gulf.

The majority (55%) of programs admit students from the medical and health fields exclusively but 13 MPH programs (45%), 8/19 in FPH and 5/10 in FM, also admit students from other fields including the social sciences.

Information on governance and curricula is available for 27 and 28 programs, respectively. Of 18 FPH-based programs, nine reported being governed by a faculty-wide committee, seven within departments, and two reported other arrangements. For nine FM-based programs, five reported being governed departmentally and four by a faculty-wide committee.

**Figure 4:**
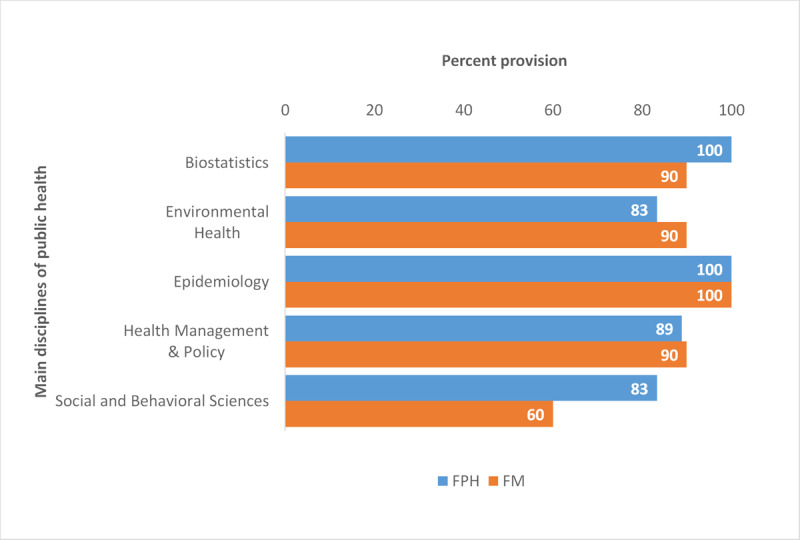
Percent of MPH programs offering courses in the main public health disciplines by Faculty, 2019.

Course offerings in the five core public health disciplines vary across programs (***Figure 4***). FPH-based programs offer more Social and Behavioral Sciences courses (83% vs. 60%) but fewer Environmental Health courses (83% vs. 90%) than FM-based programs. Offerings are largely similar in Epidemiology (100% in both), Health Management (89% FPH vs. 90% FM) and Biostatistics (100% FPH vs. 90% FM).

General MPH (i.e., with no concentration) is more common in FM-based programs (60% vs. 28%) (***Table 3***). Epidemiology, alone or with Biostatistics, and Health Management and Policy are the two most frequently offered concentrations at both FPH and FM, while relatively more FPH offer a Health Promotion/Health Education concentration.

**Table 3:** Number and percentage of public health concentrations offered by MPH programs by Faculty, 2019.


CORE PUBLIC HEALTH DISCIPLINE OR OTHER	FACULTIES OF PUBLIC HEALTH (N = 18)		FACULTIES OF MEDICINE (N = 10)		TOTAL (N = 28)	

	NO.	%	NO.	%	NO.	%

General MPH (no concentration)	5	28	6	60	11	39

Epidemiology alone or with Biostatistics	11	61	4	40	15	54

Health Management and Policy	8	44	5	50	13	46

Health Promotion/ Health Education^a^	8	44	1	10	9	32

Environmental and/or Occupational Health	6	33	2	20	8	29

Food and Nutrition	3	17	0	0	3	11

Reproductive Health/Maternal and Child Health	2	11	1	10	3	11

Tropical Health/Medical Entomology	2	11	0	0	2	7

Other fields^b^	4	14	1	10	5	18


^a^ Includes Health Promotion, Community Health, and Health Education alone or in combination. ^b^ Such as adolescent and school health, family medicine practice, geriatric health, mass gatherings (*hajj/umrah*), mental health, precision health, public health genetics and public health nursing.

**Figure 5:**
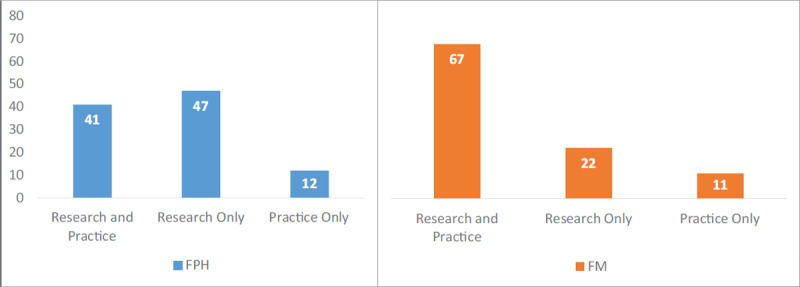
Percent of MPH programs^a^ with research and/or pratice requirements by Faculty, 2019. ^a^ Data from 26/28 completed fact sheets excluding three faculties at University of Jordan and Qatar University where information did not easily fall into our categorization, and Mogadishu University for which we do not have information.

Research and practice program requirements vary, with 10/26 research-focused requiring a research thesis or project, 3/26 practice-focused requiring a field internship or practicum, and 13/26 requiring both. There is no difference between FPH- and FM-based programs in research focus with or without a practicum (88% vs. 89%), while more FM-based programs focus on practicum training with or without research (53% vs. 78%) (***Figure 5***).

Twenty-six programs reported registration with the relevant ministry of higher education; five programs reported national accreditation. Only three, all at FPH, reported international accreditation by US (Council on Education for Public Health) or European (Agency for Public Health Education Accreditation; High Council for Evaluation of Research and Higher Education) agencies.

## Discussion

This is the first paper to map and describe university-based MPH programs over time, and it identified 19 FPH- and 10 FM-based programs across 22 Arab countries. The paper focuses on MPH as a multidisciplinary professional degree that reflects the highest commitment to public health education, equipping students with the skills and knowledge from core disciplines to address public health issues comprehensively within a health-determinants framework.

### Geographic distribution of MPH programs

Alarmingly, 10 out of 22 Arab countries lack MPH programs, which is a critical gap requiring further in-depth studies. We expected more populous countries and those with lower indices of development to offer more MPH programs to meet higher needs for public health workers, but, surprisingly, we found no correlation. The average number of MPH programs is 7 per 100 million population in the 22 Arab states, with a wide variation even among the 12 countries that offer MPH programs (range 2–73 per 100 million). It is noteworthy that the two countries with the highest number of programs per 100 million are Lebanon (73 per 100 million) and oPt (64 per 100 million). The former could be attributed to the dominance of private higher education and the absence of national planning. It is more difficult to interpret the finding for oPt but it could be related to lack of national planning and the difficulty in mobility within the occupied West Bank and East Jerusalem which encouraged local universities to offer their own programs. Egypt presents the other extreme with only two programs for 100 million population. No optimal target is recommended for this indicator and few studies have reported data that allow its computation. Globally, the US probably presents the highest ratio, with 198 accredited MPH programs or 60 programs per 100 million population using World Bank population data in 2019. In China, Hou et al. reported the number of higher education institutes for public health at 11.3 per 100 million in 2012 [19]. In India, a scoping review [5] reported that there were 44 MPH programs in 2016–17 translating to 3.29 per 100 million assuming a population of 1.339 billion in 2017 [20].

Several factors might explain these observations. The first is the country context. The first three FPH-based MPH developed in different contexts [6]. AUB, a liberal education and private university founded in 1866, was influenced by the US model of graduate public health education in establishing the first standalone school of public health in the region in 1954 (MPH with regional focus initiated 1971) [7]. The University of Alexandria, a public university, established the High Institute of Public Health in 1956 (MPH initiated 1968) under the auspices of the Ministry of Health [8]. Birzeit University’s Institute of Community and Public Health (established 1978, MPH initiated 1996) emerged from the Palestinian social action movement to address the health implications of the Israeli military occupation [9]. Gulf countries were latecomers, initiating MPH programs after 2010, also influenced mainly by the American public health education model; at least one, the MPH program at Gulf Medical University in UAE, developed in collaboration with a US school of public health. The Maghreb countries were influenced by the higher education systems of former colonial powers, namely France and Italy, and these countries did not establish independent faculties of public health or offer an MPH until recently.

International assistance is a second factor. Somalia’s three MPH programs emerged with direct Swedish aid and international support from humanitarian agencies to train local public health professionals to address the health impacts of famine and war [10]. The World Health Organization (WHO) Regional Office for the Eastern Mediterranean supported the initiation of the first MPH in the region, in 1968 at the University of Alexandria to train professionals from Egypt, as well as Arab and African countries [26]. The MPH program at Mauritania’s University of Nouakchott started in 2008 with Spanish aid [11].

The third factor, which could have influenced the establishment of standalone FPH or governance of MPH programs, is the authority of the medical profession and education. Jordan and Mauritania only offer FM-based programs and have no standalone FPH. At UAE University [28], and Université Saint Joseph in Lebanon [12], the MPH programs are offered by institutes under FM. At Sudan’s Ahfad University [13], the MPH program is housed at a standalone FPH but that is still jointly run with FM. The Kuwait University Health Sciences Center provides an interesting case where FM continues to offer an MPH (initiated 2013) even after the university established a standalone FPH (2013) with its own MPH (initiated 2018) [31]. Resistance of FM to the establishment of standalone FPH has been observed in the US and elsewhere [32–33].

### Growth of MPH programs

The MPH programs at the University of Alexandria in Egypt and AUB in Lebanon were the sole programs in the region for 20 years. FPH-based programs grew in number from one in 1968 to five over four decades (1970–2009), and then rose to 18 in one decade (2010–2019). FM-based programs grew more steadily at almost two programs every five years from 1999 until 2019. The observed growth is probably a sign of recognition of the field of public health and the need for it in the region [14].

Globally, public health education was a latecomer compared to education for the other health professions. In the US, only 13 schools offered degrees in public health in 1921–22 with only 12 accredited schools by 1960 [15]. Today in the US, there are 67 CEPH-accredited standalone schools of public health and 131 accredited MPH programs, mostly in FM [16]. There are similar trends in Europe [17], Asia [38–39], Africa [18], and Latin America [19].

In the Arab region, encouragingly, students from low socioeconomic backgrounds can pursue MPH education in the 12 countries with programs, since 16 out of 29 MPH programs are hosted at public universities, and at least one public university in each country offers an MPH. Some MPH programs at private universities may be offering scholarships or financial aid.

### Features of MPH programs

A substantial proportion of programs (50% FPH; 40% FM) reported interfaculty governance, while the rest reported being governed departmentally. Interfaculty governance may reflect the adoption of a multidisciplinary approach to the MPH but a closer study of governance dynamics and the relationship between departments is needed to validate this hypothesis.

The curricular content of FPH- and FM-based programs is more similar than expected. Both FPH and FM programs seem committed to building quantitative skills in Epidemiology (100% both) and Biostatistics (100% FPH, 90% FM) and in Health Management and Policy (89% FPH, 90% FM). Surprisingly, more FM than FPH programs offer Environmental Health (90% vs. 83%), a critical field in a region facing environmental deterioration and climate change. Social and Behavioral Sciences, essential for understanding and acting on the social determinants of health, have found their way into MPH curricula, but less so in FM than in SPH (60% vs. 83%).

Differences between FPH- and FM-based programs are clearer regarding concentrations offered and the balance between research and practice. While most FM programs are general (60%), only 20% of FPH programs are general. The reason for this may be that many FM consider the MPH a generalist second degree, complementing a basic degree in medicine or other health fields while FPH offer MPH students concentrations that define their primary professions. The two most frequently offered concentrations in both sets of faculties are Epidemiology, alone or with Biostatistics (61% FPH, 40% FM) and Health Management and Policy (44% FPH, 50% FM). More FPH programs now offer a Health Promotion/Health Education concentration (44%) indicating rising interest and career opportunities. The Environmental Health concentration receives the least attention (33% FPH, 20% FM), possibly reflecting that environmental studies are considered the purview of schools of sciences and engineering. Both FPH and FM offer MPH concentrations outside the traditional core disciplines (39% FPH, 20% FM), likely in response to perceived needs. Examples are public health nutrition, reproductive health, maternal and child health, mass gatherings, and public health genetics. AUB’s MPH program offers to students on the MPH a certificate in “Public Health in Conflict and Protracted Crises.” However, there is no full MPH concentration in this area offered by any institution in the region, signaling a gap in a region burdened by conflicts.

Surprisingly, FM programs offer more practice opportunities (63% FPH, 78% FM) while programs expose students equally to research training (88% FPH, 89% FM). We expected the contrary - that FM programs would be more interested in building research skills while FPH-based programs would be more practice- and community engagement-oriented. This finding may reflect divergent definitions of practical training, and national requirements of higher education whereby a thesis needs to be required for formal registration of a master’s degree.

Also surprising was that more FM programs than FPH programs (50% vs. 45%) allow applicants from non-health fields. Assuming a more diversified faculty and a higher interest in offering a comprehensive and multidisciplinary MPH, we expected a much higher proportion of FPH-based programs to diversify the pool of its applicants. A better indicator, i.e. the diversity of students actually admitted into these programs, is not available.

More in-depth analysis of the history and mission of such programs, as well as their admission criteria, is needed. Of particular interest is an analysis on whether a policy exists on preference of students from health or non-health backgrounds and the history and reasoning behind such a policy. In principle, MPH programs must ensure that their graduate outcomes reflect a commitment to the principles of justice and equity and a full understanding of the social and political determinants of health. Whether MPH students coming from a health/clinical background engage with these principles differently than students coming from a non-health background, such as economics or sociology, should be examined. Personal experiences by the authors show that a diverse MPH student body with a mix of health and non-health backgrounds enriches class discussions.

Understanding the educational strengths and weaknesses of MPH programs requires further study of the content and quality of education, attention to the social and political determinants of health, and faculty and student profiles. This requires information about: course syllabi; the number, qualifications, and research interests and productivity of faculty members; the quality and background of enrolled students; the student-to-faculty ratio, pedagogy, community engagement, impact, and more. Using accreditation as a surrogate indicator of quality and in the absence of a regional MPH accreditation body, we found that only three programs were accredited by US and European agencies, while a few other programs reported preparing for such accreditation. Also missing is a formal network of institutions that offer MPH programs, as exists in other regions, namely North America [20], Europe [21], Asia [22], Africa [23], and Latin America [24], and that promotes common standards in graduate public health education.

### Public health workforce

It is difficult to know how well MPH programs respond to public health workforce needs in this region in the absence of regional or global targets for the density of public health professionals per population, which by contrast are available globally as recommended targets for physicians, nurses, dentists, or pharmacists. We estimate that regional MPH programs produce 14.1 graduates per 10 million population per year. While our estimates exclude graduates of MPH programs from non-university settings, outside the region, and online programs, even a higher number of total graduates per year would not be expected to meet the vast regional public health needs. We need studies on the optimal production capacities versus needs, but also on the size and qualifications of the public health workforce in each country, including what proportion of those in public health jobs have received formal public health education, particularly MPH. FPH and FM programs can shed light on this issue by gathering information on the employment of their recent MPH graduates and alumni.

### Limitations

Our search was comprehensive. We could have missed MPH programs initiated before January 2020, but this is unlikely as we used multiple search and outreach methods. We collected basic information about MPH programs, largely from websites, but these were validated by the academic leaders at the respective faculties. We did not collect information on program history and development, resources, faculty and staff, and the nature of public health practice experiences. These are important program facets that further studies should explore.

## Conclusion

The number of MPH programs has increased in the Arab region in the last two decades, but inconsistently across countries and sub-regions with 10 out of 22 countries (45%) still without MPH programs. Our immediate aim is to use knowledge from this study to advocate with regional bodies for the establishment of a collaborative network between MPH programs at both FPH and FM in the Arab region, with the potential to expand to other countries in the WHO’s Eastern Mediterranean Region. Such a network would serve as a platform to share and exchange experiences and statistical information, develop criteria for MPH education that are sensitive to regional context and global advancements, consider the need for a regional MPH accreditation body, and advocate for more MPH programs, especially at standalone FPH, which will increase the autonomy and legitimacy of the field as a standalone profession and not a medical specialty. The eventual intent is to work together towards transforming this network into an Association of Schools/Programs of Public Health in our region learning from the experiences of such associations in other regions of the world and linking to them for global conversations on public health education and training.

## Additional File

The additional file for this article can be found as follows:

10.5334/aogh.3297.s1 Appendix 1. MPH Facts Sheet Used for validation.
