# Supplementary figures and images for: Mapping University-Based Master of Public Health Programs in the Arab world
Source: Ann Glob Health. 2021 Jul 20;87(1):70. doi: 10.5334/aogh.3297 (PMC8300585; doi:10.5334/aogh.3297)

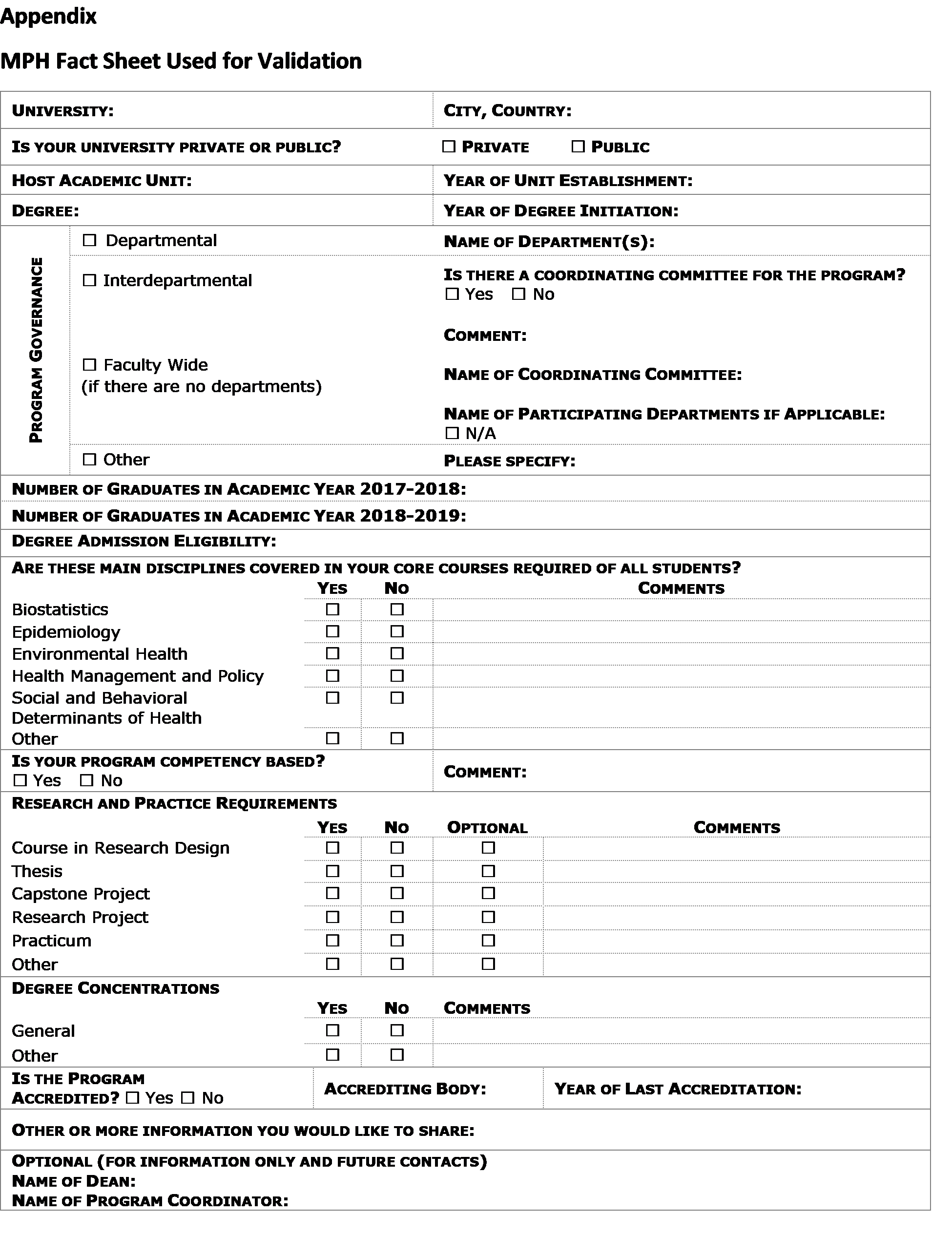

Supplement: Appendix 1. — MPH Facts Sheet Used for validation. [file agh-87-1-3297-s1.png]
